# Teratogenicity of Ochratoxin A and the Degradation Product, Ochratoxin α, in the Zebrafish (*Danio rerio*) Embryo Model of Vertebrate Development

**DOI:** 10.3390/toxins8020040

**Published:** 2016-02-05

**Authors:** Mehreen Haq, Nelson Gonzalez, Keenan Mintz, Asha Jaja-Chimedza, Christopher Lawrence De Jesus, Christina Lydon, Aaron Z. Welch, John P. Berry

**Affiliations:** 1 Department of Chemistry and Biochemistry, Florida International University, 3000 NE 151^ST^ Street, North Miami, FL 33181, USA; mhaq001@fiu.edu (M.H.); ngonz040@fiu.edu (N.G.); kmint001@fiu.edu (K.M.); ajaja001@fiu.edu (A.J.-C.); cdeje003@fiu.edu (C.L.D.J.); chlydo@fiu.edu (C.L.); 2 Chaplin School of Hospitality and Tourism Management, Florida International University, 3000 NE 151^ST^ Street, North Miami, FL 33181; USA; aawelch@fiu.edu

**Keywords:** mycotoxins, ochratoxin A, ochratoxin α, teratogenicity, zebrafish, detoxification

## Abstract

Ochratoxins, and particularly ochratoxin A (OTA), are toxic fungal-derived contaminants of food and other agricultural products. Growing evidence supports the degradation of OTA by chemical, enzymatic and/or microbial means as a potential approach to remove this mycotoxin from food products. In particular, hydrolysis of OTA to ochratoxin α (OTα) and phenylalanine is the presumptive product of degradation in most cases. In the current study, we employed the zebrafish (*Danio rerio*) embryo, as a model of vertebrate development to evaluate, the teratogenicity of OTA and OTα. These studies show that OTA is potently active in the zebrafish embryo toxicity assay (ZETA), and that toxicity is both concentration- and time-dependent with discernible and quantifiable developmental toxicity observed at nanomolar concentrations. On the other hand, OTα had no significant effect on embryo development at all concentrations tested supporting a decreased toxicity of this degradation product. Taken together, these results suggest that ZETA is a useful, and highly sensitive, tool for evaluating OTA toxicity, as well as its degradation products, toward development of effective detoxification strategies. Specifically, the results obtained with ZETA, in the present study, further demonstrate the toxicity of OTA, and support its degradation via hydrolysis to OTα as an effective means of detoxification.

## 1. Introduction

**Figure 1:**
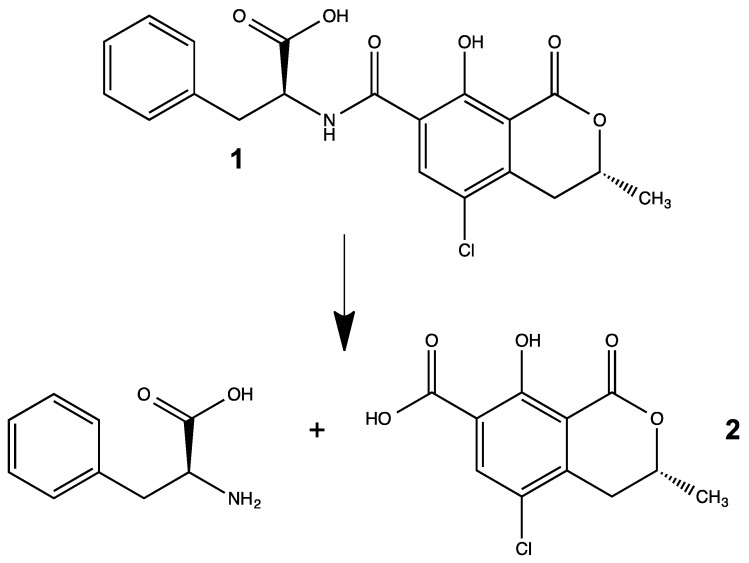
Hydrolysis of the amide bond in OTA (**1**) to yield OTα (**2**) and phenylalanine as considered the primary degradation/detoxification pathway for ochratoxins.

Ochratoxins are a family of mycotoxins produced by several fungal genera, including *Penicillium* and *Aspergillus*, and associated with contamination of various foods (e.g., fruits, grains, meats) and other agricultural products (e.g., wine, coffee) [1]. In particular, ochratoxin A (OTA; Figure 1) is, by far, the most prevalent, and generally considered the most toxic, variant of the family [2]. Structurally speaking, OTA is a chlorinated dihydroisocoumarin specifically linked, via an amide-bond, to phenylalanine. Toxicologically, OTA has been particularly linked to nephrotoxicity, and although found to a lesser extent in other tissues, it has been suggested [3,4] that specific transporters may be involved in the high accumulation of the toxin in kidneys. Consequently, exposure to OTA manifests in a recognized and well-described nephropathy, particularly in certain livestock [5]. Several studies have implicated oxidative stress in the toxicity of OTA including, in particular, a role of the nuclear factor, Nrf2 [4,6,7]. In addition to nephrotoxicity, however, other studies have indicated that OTA is carcinogenic via both genotoxic and non-genotoxic mechanisms [4,8,9,10,11]. Notably, ochratoxins have been proposed to be involved in so-called Balkan Endemic Nephropathy, an interstitial nephritis—progressing to cancer in about 50% of cases—that is limited geographically to rural farming communities along the Danube River [12,13,14]. Despite demonstrated toxicity in various animal models with respect to nephrotoxicity and carcinogenicity, a link to human health remains, in fact, unconfirmed [4].

Several recent studies have investigated the possibility of chemical, enzymatic or microbial degradation of OTA as a means of detoxification in food and agricultural products [15]. It has been observed, for example, that OTA (as a contaminant of grains and other feeds) is effectively eliminated by microflora—and particularly protozoans—of ruminants, and is not, consequently, found in milk of these exposed animals [16], whereas non-ruminants, including humans, can transfer the toxin to milk [17,18]. These and other studies, therefore, suggest a role of microbial degradation of the toxin; in fact, numerous studies have identified OTA-degrading bacteria, fungi and protozoans as recently reviewed by Abrunhosa *et al.* [19]. Specifically, the primary pathway of enzymatic degradation expected and, indeed, observed in studies [19,20,21] is the hydrolytic cleavage of the amide bond within OTA, resulting in the production of so-called ochratoxin α (OTα), and phenylalanine (Figure 1). Several studies have indicated that OTα is relatively less toxic in various models including both cell-based [22] and animal models [21], and suggest that this pathway for chemical degradation of OTA represent an effective means of detoxification. 

The present study specifically employed the zebrafish (*Danio rerio*) embryo, as a vertebrate model of teratogenicity (*i.e.*, developmental toxicity), to evaluate both OTA and its presumptive degradation product, OTα. The zebrafish system has emerged as an important toxicological model, and embryonic stages, in particular, present numerous scorable endpoints (e.g., morphological deformities, hatching, behavioral responses) relevant to evaluation of teratogenicity. Accordingly, the zebrafish embryo toxicity assay (ZETA) has been effectively utilized in various forms to investigate a wide range of environmental contaminants, including microbial toxins [23]. Moreover, as a well characterized vertebrate system, ZETA can, thereby, be employed as a proxy for understanding potential toxicity to higher animals including humans. As such, evaluation of the teratogenicity of OTA and OTα represents a step toward further understanding the proposed microbial/chemical degradation pathway as a means of detoxifying ochratoxins.

## 2. Experimental Section

### 2.1. Chemicals

Ochratoxin A was purchased from a commercial source (Sigma-Aldrich, St. Louis, MO, USA). Ochratoxin α was synthesized by hydrolysis of OTA as modified from Xiao *et al.* [24]. Briefly, OTA (1.7 mg) was refluxed in 6 N HCl (6 mL) for 72 h, and liquid-liquid extraction with chloroform was used to recover the product (0.5 mg of presumptive OTα) of the hydrolysis. The hydrolysis reaction was followed by normal-phase thin-layer chromatography (TLC) using toluene/EtOAc/90% formic acid (5:4:1) as a solvent system [25], and fluorescence under long-wave UV (*i.e.*, 366 nm) for detection; the identity of OTα was subsequently confirmed by previously described HPLC-FL and LC-MS methods [26,27] using a commercially available analytical standard of OTα (Romer Labs, Union, MO, USA). All other chemicals and reagents were purchased from either VWR Scientific (Radnor, PA, USA), or Sigma-Aldrich (St. Louis, MO, USA).

### 2.2. Zebrafish Embryo Toxicity Assay

The zebrafish embryo teratogenicity assay (ZETA) was generally conducted as previously described [23]. Briefly, chemical treatments (as MeOH solution in minimal volumes), along with solvent (*i.e.*, MeOH) controls, were added in triplicate to wells of 24-well polypropylene plates (Evergreen Scientific, Los Angeles, CA, USA), and allowed to evaporate to dryness. Subsequently, 1 mL of E3 medium [28] was added to each well, and five zebrafish embryos (≤2 hours post-fertilization [hpf], 4–32 cell stage) were transferred to each test well. Compounds were tested at a range of concentrations (based on preliminary studies) from 0.05 to 2.5 µM; specifically, OTA was tested at 0.05, 0.1, 0.25, 0.5, 1, 2 and 2.5 µM, whereas OTα was tested at 0.1, 0.25, 0.5, 1 and 2.5 µM due to relatively limited amount of compound. Embryos were subsequently observed daily using a dissecting microscope until 5 days post-fertilization (dpf) when hatching of eleutheroembryos was typically completed, and prior to larval stages (*i.e.*, ≥7 dpf). All breeding and bioassays involving zebrafish were conducted under protocols approved by the FIU Institutional Animal Care and Use Committee (IACUC), and performed by trained investigators.

### 2.3. Data Analysis

Percent mortality was calculated for each replicate (*n* = 3), and relative teratogenicity was, likewise, calculated as the percent deformed embryos per total number of live embryos in each replicate, for both OTA and OTα at each test concentration (see Section 2.2.) and each daily observation time point (1, 2, 3, 4 and 5 dpf). Relative mortality and developmental toxicity at each observational time point was calculated, respectively, as concentration for 50% lethality (LC_50_), and effective concentration (EC_50_) for teratogenicity, using GraphPad software (La Jolla, CA, USA). Relative (*i.e.*, average) mortality and development toxicity at each concentration and observational time point was evaluated by analysis of variance (ANOVA) using AnalystSoft software (Walnut, CA, USA) to determine statistical significance relative to negative (*i.e.*, untreated embryo) controls.

## 3. Results and Discussion

### 3.1. Synthesis of OTα by Hydrolysis of OTA

**Figure 2:**
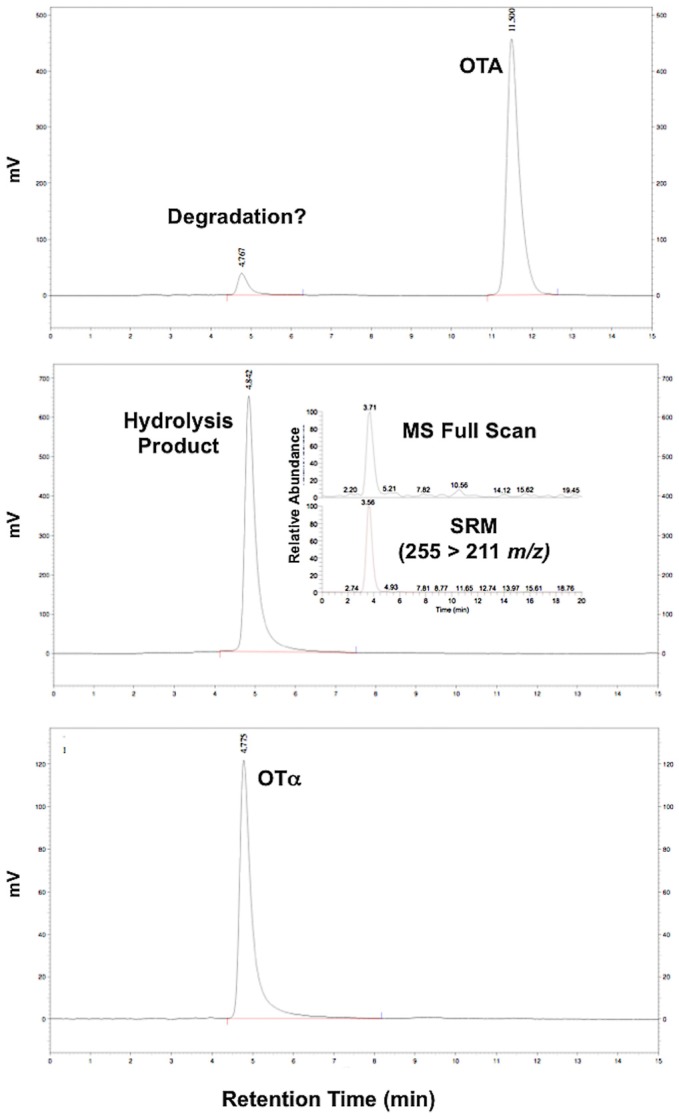
Chromatogram (HPLC-FL) of the hydrolysis of OTA to OTα. Shown are OTA standard (**A**), and subsequent hydrolysis product (**B**); for comparison, the chromatogram of the reference standard of OTα (**C**) as the presumptive hydrolysis product is shown. The identity of hydrolysis product was confirmed as OTα by LC-MS (see inset for pane (**B**)) based on the molecular ion (MH^+^ 255 *m/z*, Full Scan) and Selected Reaction Monitoring (SRM) mass transition (255 > 211 *m/z*). Apparent degradation to OTα was also observed in the OTA standard (see (**A**)).

Ochratoxin α as the presumptive degradation product of OTA was synthesized by the previously described hydrolysis [24]. The reaction was evaluated by HPLC-FL and LC-MS to confirm hydrolysis (and presence of OTα. These analyses suggest essentially complete hydrolysis of OTA to OTα (Figure 2). The identity of the hydrolysis product was confirmed by mass spectrometry (*i.e.*, LC-MS), specifically based on the observation of the OTA molecular ion (MH^+^ 255 *m*/*z*), and mass transition of the precursor to daughter (255 > 211 *m*/*z*), as well as comparison to a reference standard of OTα (Figure 2). Notably, a small amount of OTα was also observed in the standard of OTA as a presumptive degradation product. The resulting OTα (*i.e.*, hydrolysis product) was used for subsequent comparative toxicology studies.

### 3.2. Teratogenicity of OTA and OTα

**Figure 3:**
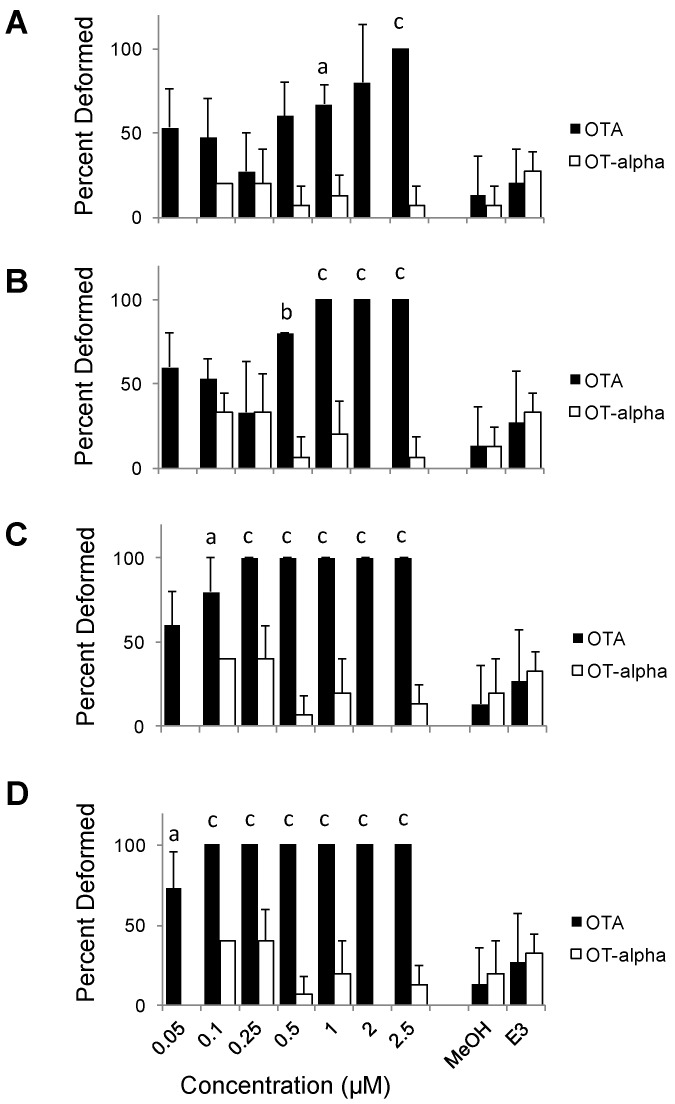
Dose and time dependence of OTA and OTα teratogenicity in ZETA. Shown are averages and standard deviations (*n* = 3) of the percent deformed embryos (5 per well/replicate) at a range of concentrations at 1 (**A**), 2 (**B**), 4 (**C**) and 5 (**D**) days post-fertilization. Activity is statistically compared to solvent (*i.e.*, “MeOH”) only controls (*n* = 3); statistically significant differences are indicated at: (a) *p* < 0.05; (b) *p* < 0.01; and (c) *p* < 0.005. No significant difference was observed between MeOH-only and untreated (“E3” *i.e.*, test medium alone) controls as shown.

**Figure 4:**
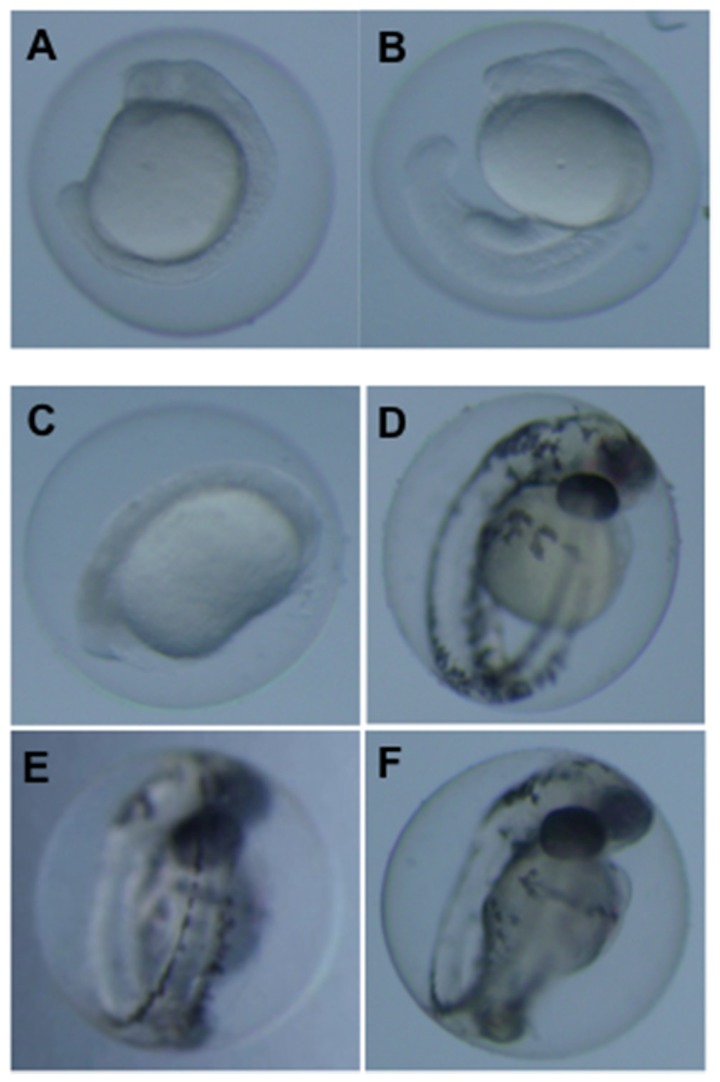
Representative development dysfunction of zebrafish embryos exposed to OTA and OTα at 1 and 3 days post-fertilization. Shown are embryos exposed to 0.5 µM OTA (**A**) compared to solvent (*i.e.*, MeOH) only controls (**B**) at 1 dpf; and embryos exposed to 0.5 µM (**C**) and 0.1 µM (**D**) OTA, and 2.5 µM OTα (**E**), compared to solvent, *i.e.*, MeOH, only controls (**F**), at 3 dpf.

**Figure 5:**
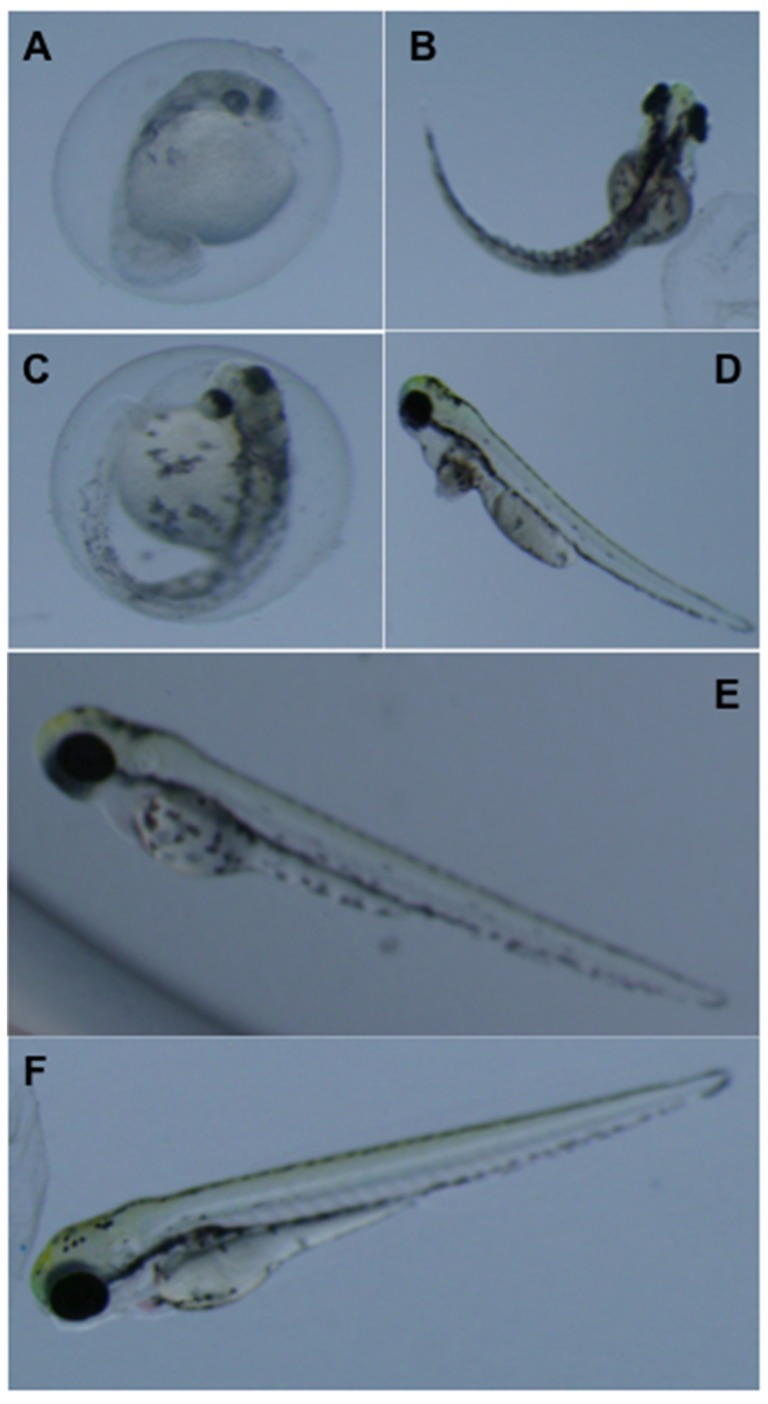
Representative development dysfunction of zebrafish embryos exposed to OTA and OTα at 5 days post-fertilization. Shown are embryos exposed to 0.5 µM (**A**), 0.25 µM (**B**), 0.1 µM (**C**) and 0.05 µM (**D**) OTA, as well as 2.5 µM OTα (**E**), compared to solvent-only (*i.e.*, MeOH) controls (**F**).

Ochratoxin A and its presumptive degradation product, OTα were evaluated for teratogenicity by ZETA. Consistent with toxicity in other models, OTA was potently toxic to zebrafish embryo development (Figure 3, Figure 4 and Figure 5). Embryos exposed to concentrations as low as 1 µM developed abnormally with frequencies significantly higher than controls by 1 dpf (Figure 3). This development toxicity increased progressively at subsequent time-points (e.g., 2, 4 and 5 dpf) observed. For example, all embryos exposed to OTA were discernibly deformed at concentrations of 1, 0.25 and 0.1 µM by 2, 4 and 5 dpf, respectively, and significant teratogenicity was, furthermore, observed at even the lowest concentration tested (0.05 µM) by 5 dpf (Figure 3). Notably, no difference was observed between 2 and 3 dpf (data not shown). Likewise, EC_50_ values for teratogenicity of OTA decreased over exposure time from 1 to 5 dpf, respectively, from 0.25 to 0.02 µM.

**Figure 6:**
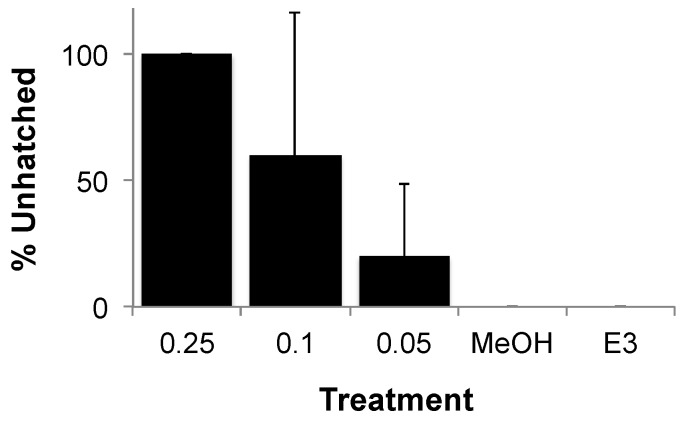
Effect of OTA on zebrafish hatching rate. Shown, for a representative exposure, is percent unhatched eggs/embryos at 5 days post-fertilization for 0.25, 0.1 and 0.05 µM OTA compared to solvent only (“MeOH”) and solvent-free medium (“E3”). At OTA > 0.25 µM, all embryos were dead or severely deformed (and not hatched). Error bars represent standard deviations (*n* = 2).

Developmental dysfunction of zebrafish exposed to OTA was characterized by discernible deformities, reduced growth and hatching rates, and lethality. At the highest concentrations (≥0.5 µM), OTA was generally lethal by 2 dpf, and development of surviving embryos was severely retarded (Figure 4). At concentrations as low as 0.05 µM, clear morphological deformities were observed by 4–5 dpf, specifically characterized by reduced growth rates, craniofacial deformities, curvature of the body axis and edemas (Figure 5), as well as a dose-dependent reduction in hatching rates (Figure 6).

Teratogenicity of OTA in the present study quantitatively and qualitatively resembles that previously observed. In prior, non-peer reviewed reports [29], developmental toxicity in the zebrafish, including edemas and malformation of fins and eyes, was observed at concentrations as low 0.6 µM and 0.2 µM by 2 and 6 dpf, respectively. More generally, dose and time dependence of teratogenicity has been previous observed in other models [2,30]. Similar to the zebrafish, OTA was found, for example, in the amphibian, *i.e.*, *Xenopus laevis*, model (*i.e.*, “Fetal Embryo Toxicity Assay *Xenopus*” (FETAX)), to be teratogenic, specifically resulting in various craniofacial deformities [2] as observed in the present study. This manifestation of developmental toxicity (*i.e.*, craniofacial deformities) has, in fact, been observed in a wide range of vertebrate models including rats [31], mice [32], hamsters [33] and chicken [30]. Studies in the FETAX system suggest EC_50_ values of approximately 60 nM compared to discernible developmental toxicity at levels <20 nM in ZETA. Moreover, the numerous practical advantages of the zebrafish model (e.g., high fecundity, completely sequenced genome, high-throughput potential, *etc.*), along with this sensitivity, suggest that ZETA is a powerful toxicological system for evaluating toxicity of OTA and its metabolites (and other degradation products).

**Figure 7:**
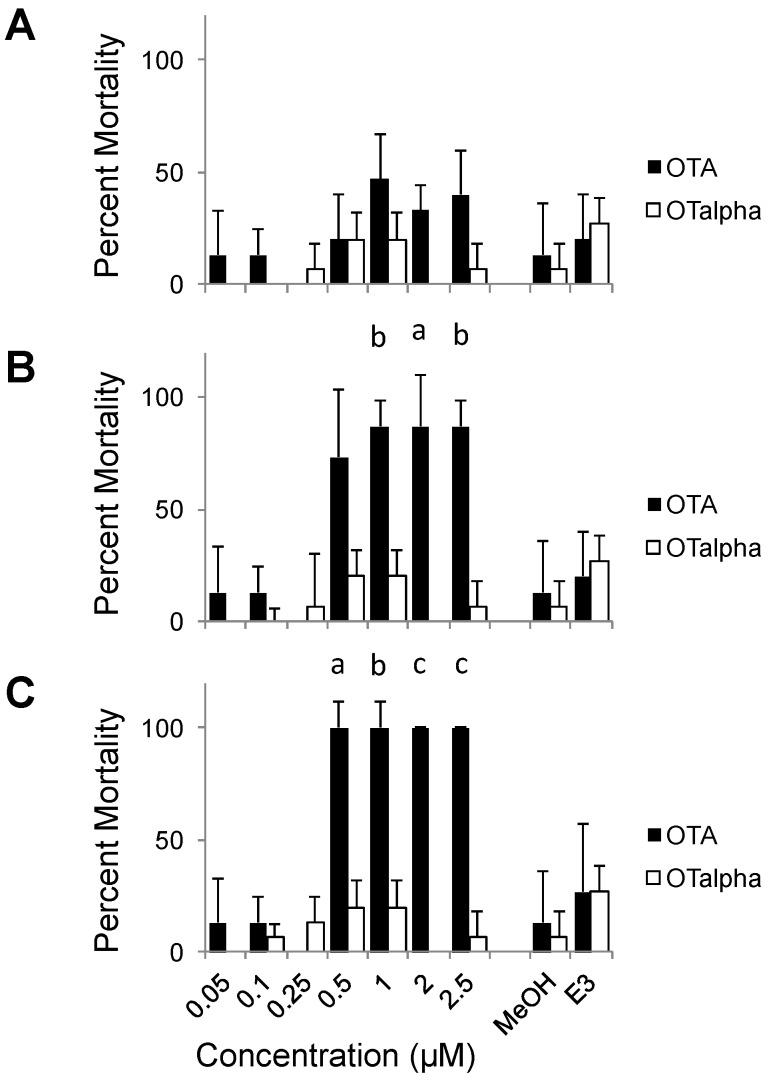
Dose and time dependence of OTA and OTα lethality in ZETA. Shown are averages and standard deviations (*n* = 3) of the percent mortality (5 per well/replicate) at a range of concentrations at 1 (**A**), 2 (**B**) and 3 (**C**) days post-fertilization. Activity is statistically compared to solvent (*i.e.*, MeOH) only controls (*n* = 3); statistically significant difference are indicated at: (a) *p* < 0.05; (b) *p* < 0.01; and (c) *p* < 0.005. No significant difference was observed between MeOH-only and untreated (*i.e.*, E3 test medium alone) controls as shown.

Compared to teratogenicity, significant lethality was observed at higher concentrations of OTA, and later stages of development (Figure 7). No statistically significant mortality was observed at 1 dpf, although slightly higher average mortalities were observed at the highest concentrations (1–2.5 µM) of the toxin. By 2 dpf, however, significant mortality among embryos exposed to OTA was observed at concentrations as low as 0.5 µM OTA. Percent mortality, however, at later stages (4 and 5 dpf) did not increase (data not shown), and significant mortality was not observed during the course of exposures at the lower test concentrations (≤0.25 µM) despite significantly higher rates of deformity in these treatments. In fact, calculated LC_50_ values were nearly an order of magnitude greater than effective concentrations (*i.e.*, EC_50_) for developmental deformities at, for example, both 1 dpf (2.14 and 0.25 µM, respectively) and 4 dpf (0.25 and 0.03 µM, respectively).

Taken together, these results generally support a high (*i.e.*, nanomolar concentration) sensitivity of zebrafish teratogenicity (compared to lethality) as a toxicological marker for OTA toxicity, and therefore, a potentially important utility of this biological system for future studies. Indeed, the observed sensitivity of ZETA (e.g., EC_50_ ≥ 20 nM) approaches the current regulatory food safety limits for OTA including, for example, European Union limits set for various agricultural and food products which are typically in the range of 1–80 ppb, *i.e.*, ~2.5 to 200 nM [34].

In contrast to OTA, embryos exposed to OTα showed neither teratogenicity, nor mortality, significantly different from that of negative controls (*i.e.*, solvent only, untreated) over 5 dpf. Generally speaking, an average of 19% frequency of deformity was observed for all concentrations tested (≤2.5 µM), and exposure/developmental times (1–5 dpf) evaluated, for OTα-exposed embryos, corresponding to <1 discernibly deformed embryo out of 5 tested per replicate (*n* = 3), which was not significantly different from either solvent (*i.e.*, MeOH) only control, or untreated, embryos (Figure 3). Furthermore, no concentration or time-dependent trends were observed for embryos exposed to OTα (Figure 3). Similar to deformity, no significantly different percent mortalities were observed over range of concentrations tested at any observational time points for OTα compared to controls (Figure 7). Taken together, these finding suggest that hydrolysis of OTA to OTα, as the presumptive degradation product of OTA (discussed below), effectively detoxifies the compound with respect to teratogenicity and mortality in ZETA.

Several previous studies have investigated the potential degradation of OTA via chemical, enzymatic or microbial metabolic means [19], specifically as a means of removing the toxin from agricultural products. Generally speaking, these studies have indicated that hydrolysis of OTA to OTα and phenylalanine (Figure 1) is the most likely, and most frequently observed, degradation pathway [19,20,21]. Recently, for example, employing the bacterial species *Cupriavidus basilensis*, Ferenczi *et al.* [21] demonstrated apparent hydrolysis of OTA to OTα, and consequent detoxification as evidenced by comparative toxicological studies in a mouse model of nephrotoxicity. Indeed, about 16 species of bacteria are known to have OTA-degrading capability, along with protozoans, yeasts and filamentous fungi and even some plants [19,20,21]. In essentially all cases in which degradation was characterized, cleavage of the amide bond to produce OTα and phenylalanine (Figure 1) was reported. The enzymes responsible for the hydrolytic cleavage have, however, varied and included, carboxypeptidases, protease and even lipases [19].

Although the present results suggest that hydrolysis of OTA to OTα is an effective means of detoxification, several studies have, in fact, suggested alternative degradation routes for OTA [19,35,36,37]. In particular, a hypothetical degradation via opening of the lactone ring has been proposed [35,37], and studies demonstrated toxicity of the open-ring OTA in a rats, although similar toxicity of the proposed degradation product in other mammal (*i.e.*, mouse) models—as well as other biological activity (*i.e.*, antibacterial)—was not observed [37]. Most recently, an amide of OTα was observed, following light and heat treatment of OTA; however, similar to OTα, the amide was also found to be generally non-toxic in cell-based assays [36]. That said, continued evaluation of metabolic products, and specifically the toxicity of the products of chemical or enzymatic degradation, is needed.

## 4. Conclusions

The results presented here suggest that the zebrafish is an effective model for evaluating toxicity of OTA and degradation products. Teratogenicity was observed at sub-micromolar concentrations with EC_50_ as low as 20 nM. This teratogenicity is quantitatively and qualitatively consistent with that observed in other animal models, but at lower effective concentrations. Along with numerous practical advantages of zebrafish, these findings support the utility of ZETA, as a rapid, sensitive and highly informative tool, for investigating toxicity of OTA and its degradative products.

Specifically, the present study confirms, as per previous studies, that OTA is acutely toxic, but its presumptive degradation product, OTα is generally not toxic. This finding, therefore, further supports these strategies for detoxifying OTA as a food contaminant. However, continued investigation of alternative degradation pathways is needed, and the ZETA system will be a useful tool to this end.
